# Design and psychometric evaluation of the breast cancer screening behaviors scale based on the health action model (HAM)

**DOI:** 10.1186/s12905-022-02026-z

**Published:** 2022-11-11

**Authors:** Fahimeh Mahboobighazaani, Masoud Karimi, Mojtaba Azadbakht, Leila Ghahremani

**Affiliations:** 1 Student Research Committee, Department of Health Promotion, School of Health, Shiraz University of Medical Sciences, Razi Ave, Shiraz, Iran; 2 Qom University of Medical Sciences, Qom, Iran

**Keywords:** Health action model, Breast cancer screening behaviors, Psychometrics, Confirmatory factor analysis

## Abstract

**Background:**

Breast cancer is known as the most common cancer and the first-rank malignancy among women. Screening plays a crucial role in early diagnosis and timely treatment. This paper presents the results of design, evaluation and analysis of a questionnaire based on the health action model to investigate breast cancer screening behaviors and their relevant factors.

**Methods:**

The study is based on using a combination of quantitative and qualitative approaches for optimal design and development of research tools. In order to design the structure of the health action model and screening behaviors related factors, both qualitative methods such as semi-structured interviews and quantitative approaches studied. Psychometric properties of the research tool were investigated through literature review. The research population consisted of 210 30–69 years old females based in Kashan who were selected through simple random sampling. The relative and content validity indexes were calculated to judge the content of the tool. Also, the confirmatory factor analysis was used to evaluate the construct validity. Additionally, intra-class correlation coefficient and Cornbrash’s alpha coefficient were calculated to evaluate the reliability of the instrument. The data were analyzed using the SPSS 22 and AMOS 22 software packages.

**Results:**

The mean age of the participants was 43 ± 9.82 Years old. The final version of the questionnaire was prepared in four sections, namely demographic factors (15 items), knowledge (12 items), constructs of the health action model or the factors related to breast cancer screening behaviors (67 items), and behavior (6 items). Factor analysis confirmed that the health action model fits in measuring breast cancer screening behaviors.

**Conclusion:**

The tool designed for measuring breast cancer screening behaviors showed acceptable psychometric properties amongst females and can be used as a valid tool in conducting research in health studies.

## Background

Breast cancer is a common type of cancer among females worldwide [1]. The burden of the disease and its mortality rate are increasing in Iranian women [2, 3], with the central and northern provinces of Iran having the highest geographical distribution of breast cancer [4]. On the other hand, it has been reported that most cases of breast cancer occur in Iranian women between the ages of 45 and 54, which shows that Iranian women are diagnosed with this cancer a decade earlier than women in developed countries [5]. Unfortunately, about 70% of patients die just a few months after starting treatment due to delay in early diagnosis, which proves the importance of diagnosis and control of this disease at the very early stages [6].

Early diagnosis of breast cancer followed by timely treatment are effective control and preventive measures that can reduce breast cancer mortality, especially in high-risk groups. Screening is a key component of the breast cancer early detection program. Early detection of breast cancer is an important step to reduce morbidity and mortality of this disease. Breast cancer screening tools include: monthly breast self-examination, clinical breast examination by a midwife/doctor and mammography [7, 8].

Several factors influence the behavior of breast cancer screening by women and the identification of these factors can play a significant role in the performance of these behaviors and thus the early detection of breast cancer. Many studies have used various tools to evaluate these behaviors and the related factors including the Breast Cancer Screening Beliefs Questionnaire (BCSBQ) developed by Kwok et al. (2016) which assesses women’s beliefs, attitudes and knowledge about breast cancer and breast cancer screening behaviors [9]. This questionnaire has been found to be a valid and reliable tool for Korean, Arab, African-Australian, Chinese-Australian and Indian women [10–13]. Another questionnaire was developed based on the protection motivation model by Khodayarian et al. (2018) to measure the effective factors in performing breast cancer screening behaviors [14]. Various other tools have also been used in different studies to explore these behaviors as well as the determinants of their performance. These studies mainly focused on single screening behaviors. A few of them assessed factors associated with a particular behavior while others ignored various aspects and effective factors including environmental, cultural and social factors [15–22].

Given that different factors alters the performance of screening behaviors for women, it is crucially important to predict screening behaviors in presence of each of these factors. Detection of the factors which are associated with such behaviors and identification of the predictors of these behaviors can be effective in the promotion of these behaviors among women. Up to now, numerous studies have employed theories and models of health education and promotion such as the Health Belief model (HBM), rational action model, theory of planned behavior, the stages of change model, social learning theory and protection motivation theory for breast cancer screening behaviors [23, 24]. Health Action Model (HAM) has also been utilized to identify the constructs that affect health and disease-related behaviors [25] This model comprehensively includes the constructs of the above-mentioned models and theories as well as various effective factors in breast cancer screening behaviors.

Also, since many factors including individual, social and environmental factors, are effective in performing breast cancer screening behaviors, it is necessary to use a comprehensive model which includes a set of these influencing factors. Therefore, in this research work the health action model was used as a guiding framework for various research stages. This model was first developed by Toons in the early 1970s to provide a framework for the professional practice of health education professionals [25]. As a selected and practical approach, this method includes the constructs of a number of models and theories and identifies key psychological, social and environmental factors which influence individual acceptance and actions related to health or illness [25].

This model consists of two main structures: 1) systems that affect behavioral intention such as belief, normative, motivational and self-concept systems and 2) factors that affect belief, normative, motivational, and self-concept systems and determine the probability of converting behavioral intentions to performance such as skills, knowledge, and environmental factors (physical, socioeconomic, and socio-cultural) [25]. Up to now, few studies have been conducted using the HAM [26–28]. However, no studies have been performed on cancer screening behaviors using this model and no comprehensive study has been conducted on three screening behaviors including breast self-examination, examination by a physician/midwife and mammography considering related factors.

In addition, there is no appropriate tool based on the HAM to measure breast cancer screening behaviors and the related factors in Iranian female population. Also there is not any tool in place capable of addressing personal and environmental factors comprehensively. Knowing that breast cancer screening behaviors are influenced by cultural, social and environmental factors that can affect people’s beliefs and practices, identifying these factors, is necessary to increase women’s participation in national breast cancer screening programs. As it is not clear which constructs of this model are better predictors of screening behaviors among females and can detect its related factors, This work tried to extract the performance of Iranian women in connection with performing cancer screening behaviors by using the methodological research approach. It also analyzed the collected data to create a suitable questionnaire based on the health action model to evaluate these behaviors. The purpose of designing this tool was to identify the factors related to performing breast cancer screening behaviors in women and to carry out effective interventions in line with these identified factors. This can lead to improved screening behaviors as an effective step in reducing breast cancer mortality.

## Materials and methods

The present cross-sectional and methodological study involved a combination of quantitative and qualitative methods in two phases of tool design and psychometrics, which was conducted in Kashan from March 2020 to October 2021.

### Tool design

**Fig. 1:**
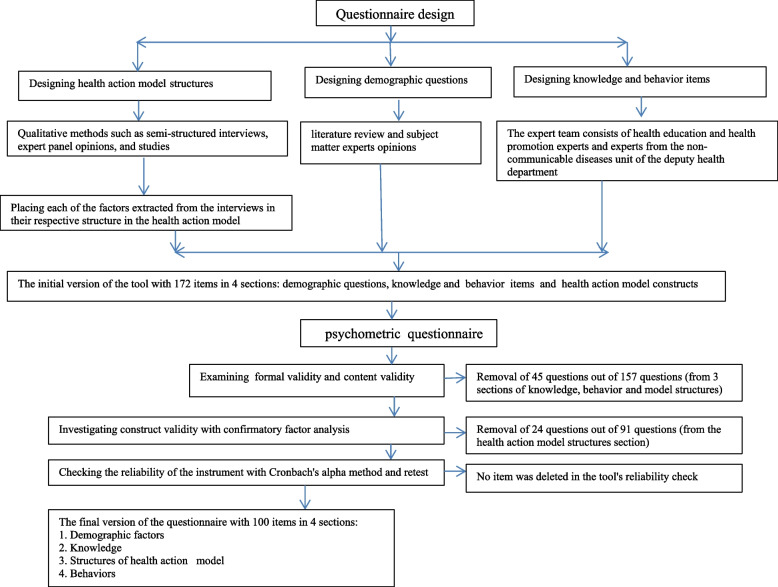
Diagram of questionnaire design and psychometrics (process of item selection)

During the design phase, different constructs of the HAM were taken into consideration for designing a questionnaire (Fig. 1).

### Designing demographic questions

The questions under this section were designed based on the conducted literature review and subject matter experts opinions.

### Designing knowledge and behavior items

To design the items of breast cancer screening behaviors and knowledge, 16 items were designed for knowledge and 6 items for behaviors with the help of a team of experts consisting of health education and promotion specialists and experts of the non-communicable diseases unit (*n* = 7).

### Designing health action model structures

To design the structures of the health action model and the factors related to performing breast cancer screening behaviors, qualitative methods such as: semi-structured interviews, opinions of expert panels and also studies conducted in this field were used [14–22] .

### Interview

Conducting interviews with women aged 69–30 were carried out based on a guided content analysis approach. At first, through consultation with professors specialized in health education and health promotion, a flexible framework was designed to formulate general questions and interview responses related to each structure of the health action model. The content of the questions used in the interview includes subjects such as: women’s knowledge and understanding of breast cancer screening behaviors, their agreement in performing these behaviors, barriers to performing these behaviors from their point of view, when and how to perform these behaviors and access to centers for performing these behaviors.

### Sampling for interviews

In the next step, sampling was conducted for interviews. The interviews were conducted with women aged 30–69 years with a directed content analysis approach. The participants were selected using purposive sampling from women living in different regions of Kashan. Totally, 22 in-depth semi-structured interviews were conducted with the participants. Qualitative data were collected until reaching data saturation and data analysis was done at the same time. The interviews lasted 20–60 minutes. The researcher and the participants agreed upon the time and place of the interviews.

### Analysis of interviews

The interviews were analyzed using directed content analysis and deductive reasoning. In this method, the HAM was the initial theory or research that existed in the field of the phenomenon and it was used as the theoretical framework. The interviews were first recorded using a recorder and were transcribed soon after. Theoretical and operational definitions were recorded for each main class that was created at the qualitative stage. The initial items were designed according to the extracted codes and the contents of the relevant classes. The extraction of the items depended on the definition of each dimension and constructs of the concept. The questionnaire items were designed to include women’s participation in breast cancer screening behaviors mentioned by the participants in their interviews. The items developed by the research team were assessed to ensure accuracy and to find overlaps and duplicates. Duplicated items were removed and those that could be merged were combined together.

Next, each of the factors extracted from the interviews was placed in its construct based on the HAM. The constructs included 11 items as follows: belief system (perceived sensitivity and severity, perceived barriers and benefits, perceived self-efficacy), normative system, motivational system, self-concept, environmental factors, skills and behavioral intention.

### The initial version of the tool after conducting a guided content analysis

The first version of the tool was prepared in four sections; i.e., demographic factors (15 items), knowledge (16 items), factors related to breast cancer screening behaviors or constructs of the HAM including perceived sensitivity (9 items), perceived severity (12 items), perceived barriers (16 items), perceived benefits (8 items), perceived self-efficacy (12 items), motivational system (11 items), normative system (25 items), self-concept (12 items), environment including physical, socioeconomic, and socio-cultural environments (12 items), skills including psychomotor skills, social interaction, and self-regulation (14 items), behavioral intention (4 items) and behavior (6 items). (a total of 172 items in 4 sections).

The knowledge section included three items receiving one score for correct answers and zero scores for false and ‘I do not know’ responses. In addition, the items of the related factors could be responded on a five-point Likert scale (strongly agree, agree, neutral, disagree, and strongly disagree). Finally, the behavior items either had two options (yes/no; items 1, 3, 4, 5, and 6) or could be scored via a Likert scale (item 2).

### Psychometric tools

During the psychometric analysis, from the indicators; face validity, content validity, construct validity and reliability were used.

### Face validity

First, the face validity of the tool was evaluated. To this end, the questionnaires were given to ten women in the target group and five specialists in the field of health education and promotion and geriatrics. These experts were asked to evaluate the level of difficulty, ambiguity, Persian grammar, use of appropriate words and placement of words in their proper places.

### Content validity (content validity ratio, content validity index)

After corrections in place based on the face validity, the questionnaires were given to 13 members of the expert team (including health education and promotion specialists, geriatrics experts, the physician in charge of the family health program, health deputy, experts of family health group, and non-communicable diseases specialists at Kashan Health Department) to assess the content validity ratio and content validity index. They were asked to categorize each question based on a three-point Likert scale with the following option: ‘the item is necessary,’ ‘the item is useful but not necessary,’ and ‘the item is not necessary.’ According to the Lawshe Table, if the number of panel members is 13 and a question is scored less than 0.62, it must be excluded from the questionnaire [29]. The method proposed by Waltz and Bausell was used to calculate the content validity index. Based on this approach, the experts were requested to determine the relevance, clarity and simplicity of each item based on a four-point Likert scale. They classified the relevance of each item as completely relevant, relevant, somewhat relevant, and unrelated, simplicity as not simple, relatively simple, simple, and completely simple, and clarity as not clear, relatively clear, clear, and completely clear. Accordingly, the items that obtained scores above 0.79 based on the minimum acceptable score in this index were retained in the test [30]. After calculating the content validity ratio and content validity index at this stage, 112 out of the 157 questions remained in the test.

### Construct validity

Next, the construct validity of the tool was assessed. The correlation matrix of knowledge, behavior, and HAM constructs was used to evaluate the construct validity of these two sections. Since the questionnaire was designed based on a model (the HAM), to evaluate the construct validity of the relevant factors, Confirmatory Factor Analysis (CFA) was employed using the AMOS 22 software. CFA helped the researcher find out to what extent the model formulated based on the theoretical framework and empirical background corresponded to reality [31, 32]. It is worth mentioning that there was no consensus on the sample size required for studies on factor analysis and structural models and the necessary minimum sample size was 200 according to many researchers [33–36], The research team chose 300 women as the minimum statistical sample. In this way, 300 women aged 30–69 from all comprehensive health service centers of Kashan University of Medical Sciences were selected by simple random sampling. The inclusion criteria were: Iranian women, living in Kashan, aged 69–30 years, having literacy and no history of breast cancer. The exclusion criteria were: women’s lack of consent to complete the questionnaire, filling the questionnaire incompletely. After obtaining the consent of the women who met the entry criteria in this age group, the women were included in the study. In addition to registering demographic information, the participants were asked to complete the instrument under study.

Out of the 300 distributed questionnaires, 206 questionnaires entered the data analysis stage due to the completeness of the information, and 94 questionnaires were excluded from the analysis due to various reasons, including incomplete information, non-response, and non-return of the questionnaires to the research team.

### Tool reliability

The reliability of the research tool was evaluated using Cronbach’s alpha coefficient and Intra-class Correlation Coefficient (ICC). The Mahalanobis Distance Index (outlier data detection index) was used to evaluate the multivariate normality and outlier data were removed. To calculate ICC, 40 participants (approximately 20% of the total participants) were retested 10 days after the first test. Alpha values and ICCs equal to or greater than 0.7 were acceptable. The high Cronbach’s alpha coefficient of the questionnaire indicated its strong reliability and accuracy in measuring each construct. According to GROVE, an alpha coefficient of 0.7 indicates the appropriate reliability of a tool [37]. For the behavior section that included questions about three screening behaviors, the Cronbach’s alpha method was not used, because each item in this section measured an independent category.

This study was performed after obtaining the ethics code (IR.SUMS.REC.1400.349) from the Vice-chancellor for Research and Technology of Shiraz University of Medical Sciences. Before starting the study, the participants were explained about the research objectives and methods and they were informed about the confidentiality of their information. They also provided consent for participation in the study and for their voices to be recorded. In addition, they were assured that the results of the project would remain confidential and all the provisions of the Declaration of Helsinki would be observed.

## Results

Out of the 300 participants in this study, 206 questionnaires were completed thoroughly without defects, and 94 questionnaires were excluded due to incomplete information and did not enter the data analysis stage. Also, during data normality checking stage, one questionnaire was excluded from the study due to having outlier data. Finally, statistical analysis was performed on 205 questionnaires. The participants’ mean age was 43 ± 9.82 years old in the psychometric phase. Other demographic characteristics of the participants in the psychometric phase have been presented in Table 1.

**Table 1:** Demographic characteristics of the participants in the psychometric stage) frequency)

Variable	Frequencies (number/percent)
**Marital status**	
Married	178 (86.8)
Single	18 (8.8)
Divorced	3 (1.5)
Widowed	6 (2.9)
**Education level**	
Illiterate	1 (0.5)
Primary school	14 (6.8)
Middle school	12 (5.9)
High school	38 (18.5)
Academic	140 (68.3)
**Occupation**	
Employee	109 (53.2)
Manual worker	5 (2.4)
Student	1 (0.5)
Freelancer	6 (2.9)
Homemaker	73 (35.6)
Other	11 (5.4)
**Insurance**	
Yes	200 (97.6)
No	5 (2.4)
**Source of health information**	
Health staff	109 (53.2)
Doctor	19(9.3)
Family	21 (10.2)
Radio and Television	20 (9.8)
Friends	8 (3.9)
Cyberspace (What-s -App, Telegram, Instagram, etc.)	20(9.8)
Other	8 (3.9)

Partial and necessary changes were made for the full clarity of the items in the study of qualitative face validity based on the reflections received from the study participants. Experts’ opinions were also applied to improve the qualitative face validity. Additionally, content validity ratio and content validity index were evaluated in order to determine the content validity of the instrument. At this stage, the number of questions decreased from 157 to 112 (4 items from the knowledge section, 4 from perceived sensitivity, 7 from perceived severity, 3 from perceived barriers, 1 from self-efficacy, 3 from motivation, 13 from subjective norms, 7 from self-concept, and 3 from skills). The results of content analysis about the constructs of the health action model along with the results of factor loading are presented in Table 3.

Considering the evaluation of construct validity in two sections of knowledge and behavior, Pearson’s correlation coefficient indicated a significant correlation between these two sections and the constructs of the HAM. Accordingly, perceived sensitivity and perceived severity had the highest correlation with knowledge and behavioral intention, while subjective norms had the highest correlation with performance. However, the constructs of the HAM were not significantly related to knowledge and performance. Additionally, self-concept was not associated with knowledge and performance. However, perceived barriers had a significant correlation with knowledge and performance. The results are presented in Table 2.

**Table 2:** Correlations between knowledge and health action, and the subscales of the health action model

Variable	S^**1**^	SE^**2**^	BA^**3**^	BE^4^	SC^**5**^	EF^**6**^	SK^**7**^	BI^**8**^	M^**9**^	E^**10**^	SN^**11**^
**Knowledge**	.414^**^	.327**	**−.123**	.396**	**.087**	.219**	.288**	.290**	.278**	.244**	.234**
**Health action**	.273^**^	^**.**^167**	−.397**	.210**	.180*	.261**	.280**	.441**	.264**	.386**	.369**

** *P* ≤ 0.001

1- sensitivity, 2- severity, 3- barriers, 4- benefits, 5- self-concept, 6- environmental factors, 7- skill, 8- behavioral intention, 9- motivation, 10- efficacy, 11- subjective norm

CFA was performed to determine the construct validity of the relevant factors assessed through Likert scales. At this stage, questions with factor loadings below 0.5 were excluded from the questionnaire. Finally, the number of questions in the questionnaire decreased from 91 to 67 in section 3 (one question excluded from perceived sensitivity, four from perceived barriers, three from perceived benefits, one from self-efficacy, three from motivation, three from subjective norms, eight from environmental factors, and one from skill). The model validity and fitness were confirmed for measuring the breast cancer screening behaviors designed based on qualitative content analysis and psychometrics. The results are summarized in Tables 3 and 4.

**Table 3:** The results of the confirmatory factor analysis of the questionnaire on breast cancer screening behaviors

Domain	Item	Load Factor	Variance Error	CVR	CVI
**Perceived sensitivity**	**1)** I may also get breast cancer.	**0.89**	**0.13**	**1**	**1**
	**2)** There is a possibility that I will get breast cancer for the rest of my life.	**0.79**	**0.27**	**1**	**1**
	**3)** As I get older, I am more likely to get breast cancer.	**0.72**	**0.43**	**1**	**1**
**Perceived severity**	**4)** If the lump in the breast is not diagnosed in time, the lump can progress quickly.	**0.52**	**0.34**	**1**	**1**
	**5)** If breast cancer is not diagnosed in time, it can cause death.	**0.67**	**0.28**	**1**	**1**
	**6)** Late detection of breast cancer is frightening.	**0.58**	**0.44**	**0.9**	**0.9**
	**7)** If breast cancer is diagnosed late, it is more difficult to treat.	**0.75**	**0.16**	**1**	**0.9**
	**8)** If breast cancer is diagnosed late, breast removal may be needed.	**0.84**	**0.12**	**0.9**	**1**
**Perceived barriers**	**9)** Fear of being diagnosed with cancer prevents screening.	**0.54**	**1.27**	**0.9**	**1**
	**10)** I do not feel good about examining my breasts.	**0.53**	**1.23**	**0.9**	**1**
	**11)** I forget to do a breast self-examination every month.	**0.61**	**0.91**	**1**	**1**
	**12)** Breast self-examination is time consuming.	**0.70**	**0.78**	**1**	**1**
	**13)** I do not know how to do a breast self-examination.	**0.59**	**1.24**	**1**	**0.9**
	**14)** Staying away from the mammography center will prevent this examination.	**0.80**	**0.58**	**0.9**	**0.9**
	**15)** I do not do mammography because it is painful.	**0.81**	**0.53**	**1**	**1**
	**16)** Prolonged waiting for a mammogram prevents this examination.	**0.81**	**0.57**	**1**	**1**
	**17)** Because I’m not insured, I do not have mammograms.	**0.73**	**0.59**	**1**	**1**
**Perceived benefits**	**18)** I feel satisfied and safe by doing a breast self-examination.	**0.65**	**0.33**	**1**	**1**
	**19)** Breast self-examination reduces the risk of dying from breast cancer.	**0.72**	**0.20**	**1**	**1**
	**20)** An examination by a midwife will reveal lumps that I have not been able to diagnose myself.	**0.81**	**0.13**	**0.9**	**1**
	**21)** Early detection of breast cancer reduces the complications of the disease.	**0.65**	**0.11**	**1**	**0.9**
	**22)** Early detection of breast cancer can significantly reduce the cost of the disease.	**0.74**	**0.19**	**1**	**0.9**
**Self-efficacy**	**23)** I’m sure I can learn the skill of breast self - examination.	**0.59**	**0.28**	**1**	**1**
	**24)** I can adjust my schedule to have a breast self-examination at least once a month.	**0.53**	**0.54**	**1**	**1**
	**25)** I can set my time so that I do not forget to do a breast self-examination on time.	**0.56**	**0.61**	**1**	**1**
	**26)** I can find a secluded and suitable place for breast self-examination.	**0.64**	**0.42**	**1**	**1**
	**27)** Even if I am embarrassed, I still go to the doctor / midwife for a breast exam.	**0.62**	**0.48**	**1**	**1**
	**28)** Despite the pain of mammography, I can have a mammogram.	**0.67**	**0.37**	**0.9**	**1**
	**29)** By managing my expenses, I can afford a mammogram.	**0.78**	**0.23**	**1**	**1**
	**30)** Despite the remoteness of the mammography center, I can have a mammogram if needed.	**0.79**	**0.22**	**1**	**1**
	**31)** Despite my busy schedule, I can still have screening tests.	**0.72**	**0.40**	**1**	**1**
**Motivation**	**32)** Maintaining and promoting health is very important to me.	**0.59**	**0.14**	**0.9**	**1**
	**33)** My health also affects my family.	**0.54**	**0.15**	**0.9**	**1**
	**34)** Reducing extra costs motivates me to have breast cancer screening exams.	**0.57**	**0.76**	**1**	**1**
	**35)** Maintaining my appearance and beauty motivates me to undergo breast cancer screening examinations.	**0.90**	**0.11**	**1**	**1**
**Subjective norm**	36) People around me (mother, sister, wife, friends) believe that I should do a breast self-examination on a monthly basis.	**0.66**	**0.51**	**1**	**1**
	37) Health professionals believe that I should have a breast self-examination on a monthly basis.	**0.68**	**0.40**	**0.8**	**1**
	38People around me (mother, sister, wife, friends) believe that I should be examined by a doctor / midwife if necessary.	**0.70**	**0.45**	**0.9**	**0.8**
	39) Health care professionals believe that I should be examined by a doctor / midwife if necessary.	**0.65**	**0.37**	**1**	**1**
	40) People around me (mother, sister, wife, friends) believe that I should have a mammogram if necessary.	**0.73**	**0.40**	**1**	**1**
	41People around me (mother, sister, family members, friends) will be examined by a doctor / midwife if necessary.	**0.61**	**0.56**	**0.8**	**0.9**
	42) People around me (mother, sister, family members, friends) do mammograms if necessary.	**0.70**	**0.37**	**0.8**	**1**
	43) For those around me (mother, sister, family members, friends), breast self-examination is a healthy behavior.	**0.80**	**0.22**	**1**	**1**
	44) From the point of view of those around me (mother, sister, family members, friends) Screening behaviors is a sign of health responsibility.	**0.75**	**0.29**	**1**	**1**
**Self concept**	45) I take great care of my health.	**0.78**	**0.21**	**1**	**1**
	46) I care about my family’s health.	**0.56**	**0.19**	**1**	**1**
	47) I am a healthy person.	**0.74**	**0.28**	**1**	**1**
	48) I have a regular plan for life.	**0.74**	**0.30**	**1**	**0.9**
	49) I value my health very much.	**0.88**	**0.12**	**1**	**1**
**Environment factors**	50) Family support (spouse, father, mother, sister) is effective in performing screening examinations.	**0.58**	**0.22**	**0.9**	**1**
	51) My family (spouse, father, mother, sister) sponsors me for a mammogram.	**0.68**	**0.40**	**0.9**	**1**
	52) The health center provides me with information support for mammography.	**0.64**	**0.45**	**0.9**	**0.9**
	53) My friends support me emotionally and informational for mammography.	**0.67**	**0.52**	**1**	**1**
**Skill**	**54)** I can make a detailed schedule for breast cancer screening tests.	**0.66**	**0.33**	**0.8**	**1**
	**55)** I have the ability to run my own programs.	**0.78**	**0.23**	**0.8**	**0.9**
	**56)** I have the ability to obtain reliable information on breast cancer screening.	**0.80**	**0.22**	**0.8**	**0.9**
	**57)** When I have a goal, I can plan to achieve it.	**0.77**	**0.20**	**0.9**	**1**
	**58)** I am able to achieve the goals I have set for myself.	**0.81**	**0.19**	**1**	**1**
	**59)** I can communicate well with healthcare staff.	**0.80**	**0.19**	**1**	**1**
	**60)** I can communicate well with the mammography technician.	**0.75**	**0.31**	**0.9**	**1**
	**61)** I can do a breast self-examination every month.	**0.72**	**0.40**	**0.9**	**1**
	**62)** I can have a doctor / midwife examination if necessary.	**0.73**	**0.24**	**1**	**0.9**
	**63)** I can have a mammogram if my doctor diagnoses it.	**0.74**	**0.19**	**1**	**1**
**Behavioral intention**	**64)** I intent to have breast cancer screening tests from now on.	**0.81**	**0.18**	**0.9**	**1**
	**65)** I intent to have a breast self-examination on a monthly basis.	**0.79**	**0.22**	**0.9**	**1**
	**66)** I intent to see a doctor / midwife immediately if I diagnose a breast lump myself.	**0.85**	**0.09**	**0.9**	**1**
	**67)** I intent to have a mammogram if prescribed by a doctor / midwife.	**0.75**	**0.18**	**0.9**	**1**

*CVR* Content Validity Ratio, *CVI* Content Validity Index

**Table 4:** Fit indices of the CFA of the questionnaire

Model fit index	Default model	Modified model
**Chi-square/degrees of freedom ratio (χ2/df)**	**3.2** ***P*** **< 0.001**	**2.2** ***P*** **< 0.001**
**Goodness-of-Fit Index (GFI)**	**.66**	**.90**
**Adjusted Goodness-of-Fit Index (AGFI)**	**.62**	**.87**
**Comparative Fit Index (CFI)**	**.59**	**.92**
**Root Mean Square Error of Approximation (RMSEA)**	**.08**	**.05**

*CFA* Confirmatory factor analysis

Values equal to or higher than 0.7 were considered acceptable to evaluate the reliability of the questionnaire using the internal consistency method and Cronbach’s alpha coefficient. ICC was used to evaluate the repeatability of the constructs and internal stability. The results are shown in Table 5.

**Table 5:** Internal consistency (Cronbach’s alpha) and ICC of the sections of the studied tool

Items	ICC	Cronbach’s alpha
**Knowledge**	.997^**^	.999*
**Sensitivity**	.882^**^	.937*
**Intensity**	.925^**^	.961*
**Benefits**	.994^**^	.997*
**Barriers**	.960^**^	.980*
**Efficacy**	.915^**^	.956*
**Motivation**	.959^**^	.979*
**Subjective norms**	**1**	**1**
**Self- concept**	.985^**^	.992*
**Environmental factors**	.971^**^	.985*
**Skill**	.952^**^	.976*
**Behavioral intention**	.985^**^	.993*
**Health action**	**1**	**1**

**p ≤ 0.001, *P <0.05

*ICC* Intraclass Correlation Coefficient

The final version of the questionnaire consisted of 100 items in four sections including demographic factors (15 items), knowledge (12 items), HAM constructs (perceived sensitivity (3 items), perceived severity (5 items), perceived barriers (9 items), perceived benefits (5 items), perceived self-efficacy (9 items), motivation (4 items), subjective norms (9 items), self-concept (5 items), environmental factors (4 items), and skills (10 items), behavioral intention (4 items), and behavior (6 items). The items related to knowledge and behavior were responded via a five-point Likert scale ranging from strongly agree to strongly disagree (Fig. 1).

## Discussion

The present study aimed to design and psychometrically evaluate a questionnaire to assess breast cancer screening behaviors and the relevant factors based on the HAM in Iranian women. The study participants included 205 women aged 30–69 years living in Kashan. Since the model covered numerous comprehensive constructs and factors, it seemed to be able to detect the factors that affected the women’s performance of breast cancer screening behaviors. Evaluation of content validity of the tool indicated that content validity ratio and content validity index were close to the desired level for most cases, representing the high validity of the questionnaire. Overall, the findings revealed the optimal validity and reliability of the tool. The results of confirmatory factor analysis also demonstrated the acceptable fit of the relevant items.

This study also assessed the applicability of the HAM for determining the participation of Iranian women in breast cancer screening behaviors and its determinants. According to the results, there was no study on the validation of the HAM and there was no suitable tool to simultaneously measure three breast cancer screening behaviors in the target group (women aged 30–69 years) to make a comparison.

According to a mixed-methods study conducted by Khodayarian et al. (2018) in Yazd that aimed to design and psychometrically evaluate a questionnaire based on the theory of protection motivation for Iranian women to participate in breast cancer preventive behaviors, all constructs of the theory of protection motivation (8 constructs) obtained an impact score higher than 1.5. The reliability of the questionnaire was also acceptable. In addition, factor analysis confirmed the fit of the theory of protection motivation for the participation of Iranian women in preventive behaviors against breast cancer. Moreover, perceived sensitivity, perceived severity, and self-efficacy constructs had acceptable factor loadings in determining the participation of Iranian women in breast cancer prevention behaviors, which was in agreement with the present study findings [14].

Norfariha Che Mohamed et al. [38] conducted another study in Malaysia to evaluate the validity and reliability of a questionnaire based on the Health Belief Model for promoting self-examination and mammography screening for early detection of breast cancer in 103 women aged 35–70 years. The results of exploratory factor analysis indicated nine factors including self-efficacy for mammography and breast self-examination, perceived barriers to breast self-examination and mammography, perceived sensitivity and severity of breast cancer, cue to action for mammography screening, perceived benefits of breast self-examination and mammography and health motivation, with 54 items accounting for 74.2% of the observed variance. The reliability of the questionnaire was confirmed too. The final questionnaire was prepared with 54 items and it was reported to help assess women’s beliefs about accepting breast self-examination and mammography in healthcare performance and research [38].

Even though these two studies designed and psychometrically evaluated two acceptable tools for investigating breast cancer prevention behaviors, the first study put much emphasis on lifestyle-related behaviors, mammography screening behaviors and physician examination However, it did not evaluate breast self-examination. The second study also emphasized the screening behaviors of self-examination and mammography, but did not assess the screening behavior of physician examination. On the contrary, all the three screening behaviors and their relevant factors evaluated in the present study. Furthermore, the two aforementioned studies put much emphasis on the constructs of the Protection Motivation Theory and the Health Belief Model, while most of these constructs were personal factors. In addition, they did not assess the external and environmental factors affecting such behaviors. However, the present study investigated more comprehensive constructs including internal factors such as beliefs, motivational factors, environmental factors affecting such behaviors such as social support and cultural factors. These were strength points of the current research work.

In this work, the results of determining the construct validity using CFA indicated that the model had a relatively good fit. This implied that the proposed model met the main hypotheses of the health action model in the target population.

Considering the construct validity in the knowledge and behavior sections, Pearson’s correlation coefficients indicated a significant correlation between these two sections and the constructs of the HAM. Accordingly, perceived sensitivity and severity constructs had the highest correlation with knowledge, while subjective norms and behavioral intention had the highest correlations with performance. Additionally, only the self-concept construct had no significant correlation with knowledge and performance. There was also a significant correlation between perceived barriers, and knowledge and performance. Regarding the correlation between perceived sensitivity, severity, knowledge and performance, people with higher levels of knowledge and awareness about health issues become more cautious and understand its importance. This will be in turn more evident in their actions and behaviors. Women who believe they are susceptible to breast cancer and feel more at risk are more likely to engage in breast cancer screening behaviors.

Considering the perceived barriers construct and its negative correlation with knowledge and behavior, it can be concluded that the higher the knowledge of individuals, the less important the barriers to behaviors and the more important the benefits of that behavior will be, which will eventually lead to their better performance. The same is true for constructs such as environmental factors, skills, motivation, self-efficacy, subjective norms and behavioral intention.

A positive consequence as well as a strength point of this tool was that the tool is designed based on the health action model which can be used as a framework for planning at the national or provincial levels. This is useful as the health and treatment sector practitioners can use the tool as a model for requirement assessment at every level and plan and implement effective interventions to improve screening behaviors based on it.

The conducted study had limitations as follows: There was no similar tool to be compared with the one developed in this study. The sample size in this study was originally 300 people, which due to the lock down and Covid-19 Pandemic reduced to 206 people. Due to the large number of questions in the questionnaire many candidates did not managed to complete the questionnaires and only 206 questionnaires were completely filled in. Based on this, it is suggested that the study to be conducted in other areas or at another time with a larger sample size in order to obtain better results and a better fit of the model.

## Conclusion

According to the results of the present study, the designed questionnaire had innovative aspects based on real cultural beliefs for assessing women’s participation in breast cancer screening behaviors. Besides, its optimal validity and reliability make it an appropriate candidate to be used in similar studies. Therefore, this questionnaire can be used as the main tool in research works based on the HAM as well as in planning for theory-based educational interventions. Since breast cancer is highly prevalent among Iranian women and breast cancer screening behaviors are a part of the national guidelines of the Ministry of Health, conducting educational interventions based on health education theories can empower women in this field and encourage them to do such behaviors, thereby reducing breast cancer mortality.

## Data Availability

The datasets used in this study are available from the corresponding author on request.

## Abbreviations

HAM: Health action model
CFA: Confirmatory factor analysis
